# Anti-Nasopharyngeal carcinoma mechanism of sanguinarine based on network pharmacology and molecular docking

**DOI:** 10.1097/MD.0000000000036477

**Published:** 2023-12-01

**Authors:** Jing-Ying Fan, Jie Liu, Wen-Qing Zhang, Ting Lin, Xi-Ran Hu, Fang-Liang Zhou, Le Tang, Ying-Chun He, Hong-Jian Shi

**Affiliations:** a Hunan University of Chinese Medicine, Changsha, China; b Hunan Provincial Engineering and Technological Research Center for Prevention and Treatment of Ophthalmology and Otolaryngology Diseases with Chinese Medicine and Protecting Visual Function, Hunan University of Chinese Medicine, Changsha, China; c Hunan Provincial Key Lab for the Prevention and Treatment of Ophthalmology and Otolaryngology Diseases with Traditional Chinese Medicine, Hunan University of Chinese Medicine, Changsha, China.

**Keywords:** apoptosis, MAPK/ERK signaling pathway, nasopharyngeal carcinoma, proliferation, sanguinarine

## Abstract

**Background:**

The purpose of this study was to investigate the mechanism of sanguinarine (SAN) against nasopharyngeal carcinoma (NPC) by means of network pharmacology, molecular docking technique, and experimental verification.

**Methods:**

The SAN action targets were predicted using the Swiss Target Prediction database, the related NPC targets were determined using the GEO database, and the intersection of drug and disease pathway targets were considered to be the potential targets of SAN against NPC. The target-protein interaction network map was constructed using the STRING database, and the core target genes of SAN against NPC were obtained via topological network analysis. “R” language gene ontology (GO) function and Kyoto encyclopedia of genes and genome (KEGG) pathway enrichment analyses were used to dock the core target genes with SAN with the help of AutodockVina. Cell proliferation was detected using MTT and xCELLigence real-time cell analysis. Apoptosis was identified via Hoechst 33342 staining, JC-1 mitochondrial membrane staining, and annexin V-FITC/PI double fluorescence staining, while protein expression was quantified using western blotting.

**Results:**

A total of 95 SAN against NPC targets were obtained using target intersection, and 8 core targets were obtained by topological analysis and included EGFR, TP53, F2, FN1, PLAU, MMP9, SERPINE1, and CDK1. Gene ontology enrichment analysis identified 530 items, and 42 items were obtained by Kyoto encyclopedia of genes and genome pathway enrichment analysis and were mainly related to the PI3K/AKT, MAPK, and p53 signaling pathways. Molecular docking results showed that SAN had good binding activity to the core target. SAN inhibited the proliferation of NPC cells, induced apoptosis, reduced the expression levels of survivin and Bcl2, and increased the expression levels of Bax and cleaved caspase-8. It also decreased the expression levels of the key proteins p-c-Raf, p-MEK, and p-ERK1/2 in the MAPK/ERK signaling pathway in NPC cells.

**Conclusion:**

SAN inhibits the proliferation and induces the apoptosis of NPC cells through the MAPK/ERK signaling pathway.

## 1. Introduction

Nasopharyngeal carcinoma (NPC) is one of the most common malignant tumors in the head and neck. Its incidence has obvious regional characteristics, mostly in southeast Asia, and a high incidence in southern China, affecting more males than females.^[1]^ Scholars believe that the occurrence of NPC is the result of a combination of factors, which is related to family genetic susceptibility, EB virus infection, environmental factors, and eating habits. The most common site of NPC is in the pharyngeal recess. Because of its hidden location and the lack of specificity of early symptoms, NPC is not diagnosed in time, its metastasis rate is high, and most of NPC cases are poorly differentiated carcinomas, making its prevention and treatment more difficult. NPC is sensitive to radiation and radiotherapy is the first choice for its treatment. Simultaneous chemotherapy can enhance the antitumor effect and reduce the metastasis and recurrence rates.^[2,3]^ However, radiotherapy and chemotherapy have limitations.^[4]^ The side effects caused by this treatment, such as dry mouth, mucosal injury, and nerve injury, result in significant levels of pain and lead to a decline in quality of life. Local recurrence and metastasis are still the main causes of failure in the treatment of NPC. More and more studies have shown that natural small molecular compounds demonstrate efficacy in the treatment of tumors with low levels of side effects and high safety. The discovery and application of these drugs are expected to provide a new treatment plan and perspective for tumor treatment. Sanguinarine (SAN) is a phenanthrene alkaloid extracted from plants, such as Macleaya corydalis and Corydalis.^[5]^ Studies have found that SAN has a wide range of pharmacological effects, which can activate the AMPK/Smad3 signaling pathway to promote MC3T1-E1 cell differentiation, stimulate bone growth, treat osteoporosis,^[6]^ inhibit activation of the P38 MAPK signaling pathway, relieve neuropathic pain,^[7]^ reduce the pathogenicity of Magnaporthe oryzae,^[8]^ inhibit the MAPK signaling pathway, and affect the release of inflammatory mediators.^[9]^At present, more and more attention has been paid to the antitumor research on SAN, which covers many aspects, such as signaling pathways and microRNAs.^[10,11]^ SAN has therapeutic effects on lung adenocarcinoma,^[12]^ breast cancer,^[13,14]^ ovarian cancer,^[15]^ thyroid cancer,^[16]^ colorectal cancer,^[17]^ and gastric cancer.^[18]^ It also inhibits the proliferation of non-small cell lung cancer, blocks the cell cycle, and induces apoptosis by changing the content of STAT3.^[12]^ In addition, its mechanism is related to ferroptosis.^[19]^ Sarkhosh-Inanlou et al have reported that SAN reverses the sensitivity of cisplatin-resistant ovarian cancer cells to cisplatin by reducing the glutathione content.^[15]^ S. Zhang et al have shown that SAN has an anti-ovarian cancer effect via the long noncoding RNA CASC2/EIF4A3 axis, NF-κB, and PI3K/AKT/mTOR signaling pathways.^[20]^ It also affects the viability of triple negative breast cancer cells through CCL2 and the IKBKE/NF-κB/ERK1/2 signaling pathway.^[13]^ In hypoxic conditions, SAN inhibits the expression of HIF-1α in hepatoma cells, further inhibiting the secretion of TGF-β and epithelial stromal transformation induced by hypoxia.^[21]^ It has been reported that SAN can inhibit the proliferation, apoptosis, and invasion of NPC cells through the mTOR signaling pathway.^[22]^ However, research on its mechanism is still not comprehensive enough, which hinders its in-depth study and clinical application.

Network pharmacology is a new strategy that integrates systems biology, high-throughput screening, and computer technology to study the complex relationship between “drug-target-disease” and accelerate the identification of drug targets. Molecular docking technique is a method to evaluate the interaction between organic small molecular ligands and target proteins, which can be used to determine the binding sites and binding forces of small molecules in natural products and target proteins.^[23,24]^ Based on this, the purpose of the present study was to use network pharmacology and molecular docking technology to explore the anti-NPC mechanism of SAN and to verify its antitumor activity and mechanism against NPC through experiments.

## 2. Methods

### 2.1. Screening of sanguinarine targets

SAN was uploaded to the pubchem BATMAN database (http://bionet.ncpsb.org.cn/ batman-tcm/index.php/Home/ Index/ index) as ID: 5154 and the target with score of > 20 was selected. The molecular structure was uploaded to the PharmMapper database (http://lilab-ecust.cn/pharmmapper/). Results with Norm Fit of >0.5 and z-score of >0 were selected, and the SwissTargetPrediction database (http://www.swisstargetprediction.ch/) was searched using “Sanguinarine” in Traditional Chinese Medicine Systems Pharmacology Database and Analysis Platform (https://tcmsp-e.com/tcmsp. php) database and the corresponding formula for its molecular structure. The above results were combined to obtain the SAN targets. The results were corrected using the reviewed target from Swiss-Prot and Human in Uniprot data.

### 2.2. NPC target screening

Transcriptome datasets related to NPC were selected from the GEO database and included GSE68799, GSE118719, and GSE134884 (Table 1). The 3 transcriptional groups were analyzed using R software v-4.1.3 package “DEseq2,” “limma.” Results with *P* value of <.05 and |log2FC| > 1 were selected as differential genes. All differential genes were merged as NPC targets. The SAN and NPC-related targets were mapped using a Venn diagram with the help of a Bioinformatics online tool (https://bioinformatics.psb.ugent.be/webtools/Venn/) to obtain the intersection target of SAN in the treatment of NPC.

**Table 1:** The number of differential genes.

GSE ID	Normal	Tumor
GSE68799	4	42
GSE118719	4	7
GSE134884	3	3

### 2.3. Screening of core targets for SAN therapy of NPC and construction of protein–protein interaction (PPI) network

Interactions between target–target function-related proteins were obtained from the mapped intersection targets for NPC SAN treatment in the STRING (version 11.5) database. The selected minimum required interaction score was >0.700 and the PPI network map of target interaction and its tsv.data were obtained. The topological parameters, such as the median degree of freedom and the maximum degree of freedom in the network, were analyzed using NetworkAnalyzer in Cytoscape_v3.7.1. The core targets were selected based on 2 times the degree.

### 2.4. Enrichment analysis of gene ontology (GO) and Kyoto encyclopedia of genes and genome (KEGG) pathways

R packages including “ClusterProfiler” (version 4.1.3) were used to analyze and visualize the GO and KEGG pathway enrichment of the module and core targets. Gene annotation information came from “org.Hs.e.g..Db” and *P*-value cutoff for enrichment was .05.

### 2.5. Molecular docking

Molecular docking can predict the binding ability of proteins to small molecules. The SAN structure was obtained from PubChem database (https://pubchem.ncbi.nlm.nih.gov/), and the corresponding protein structure was extracted from the PDB database (https://www.rcsb.org/). The SAN structure was optimized using minimum energy MM2 position 3D Draw module in Chem Bio Office software and transformed into PDBQT using Raccoon software. Autodock Vina software-supporting tool MGLTools 1.5.6 was used to evaluate the proteins, hydrogenation, and Gasteiger charge, as well as merge nonpolar hydrogen and to perform other operations. The results were saved in pdbqt format to prepare for ligand docking.

### 2.6. MTT assay

The cells were resuspended at a concentration of 5 × 10^4^ cells/mL and 100 μL/well of the cell suspension were seeded into a 96-well plate. After the cells were attached, 200 μL of blood root alkaloids at different concentrations was added. After the NPC cells were treated with the drug for 12, 24, 36, or 48 hours, the culture medium was removed and 100 μL of the MTT working solution was added. The cells were then kept in an incubator for 3 hours, after which the supernatant was removed, 100 μL of dimethyl sulfoxide was added, and the solution was crystallized. The absorbance value at 490 nm was determined using an automatic enzyme labeling instrument.

### 2.7. xCELLigence real-time cell analysis (RTCA)

First, 50 μL of complete culture medium were added to the cell culture E-plate, which was placed on the RTCA instrument to measure the baseline. The cells were digested and resuspended at a concentration of 5 × 10^4^ cells/mL. Then, 100 μL/well of the cell solution was added to the culture E-plate and incubated for 20 minutes. The culture E-plate was then placed in the RTCA instrument to monitor cell proliferation. Different concentrations of drugs were added before the cell proliferation curve entered a plateau and the cells were continued to be monitored for more than 48 hours.

### 2.8. Hoechst 33342 staining

The cells were digested, resuspended at a concentration of 1 × 10^5^ cells/mL, and 1 mL/well of the cell suspension was added to a six-well plate. After the cells were attached, 2 mL of the drug-containing culture medium was added. The culture medium was removed after 24 hours and washed with phosphate-buffered saline (PBS) twice. Then, 1 mL of Hoechst 33342 staining solution was added and incubated for 20 minutes in the dark. Next, the staining solution was removed, and the excess dye was washed with PBS. Finally, the cells were imaged using a multi-functional cell imaging system.

### 2.9. JC-1 mitochondrial membrane staining

The cells were digested, resuspended at a concentration of 1 × 10^5^ cells/mL, and 1 mL/well of the cell suspension was added to a six-well plate. After the cells were attached, 2 mL of the drug-containing culture medium was added. The culture medium was removed after 24 hours and washed twice with PBS. Then, 1 mL of the JC1 staining solution was added and incubated for 20 minutes in the dark. The staining solution was then removed and the excess dye was washed out with PBS. The cells were imaged using a multi-functional cell imaging system.

### 2.10. Annexin-V FITC/PI staining

After the intervention with SAN, the supernatant was collected in an Eppendorf (EP) tube and the adherent cells were digested with trypsin without EDTA. Then, 2 mL of the whole culture was added to terminate the digestion, the cell suspension was collected in an EP tube containing the supernatant, and the supernatant was discarded and transferred to a new 1.5-mL EP tube with PBS solution. The supernatant was removed by centrifugation and 100 μL of binding buffer was gently added, followed by 5 μL of annexin V-FITC and 5 μL of propidium iodide (PI) staining solution while avoiding light exposure for 15 minutes. Then, 100 μL of binding buffer was added to stop the staining reaction. The apoptosis rate was detected using a double fluorescence cell analyzer.

### 2.11. Western blot

After SAN treatment for 24 hours, the total protein was extracted and quantified. The protein samples were then added to the gel electrophoresis system, concentrated at a voltage of 40 V in the gel, and pressurized to 80 V after entering the separation gel at 200 mA of constant current. 2.5 hours transfer film and 5% skimmed milk powder closed for 1 hour. The primary antibody was incubated overnight at 4 ºC, and the secondary antibody was incubated at room temperature for 1.5 hours. The film was scanned using a two-color infrared fluorescence imaging system, and the band signal value was analyzed using ImageStudio.

### 2.12. Statistical analysis

SPSS 26.0 statistical software was used to process all of the data in the present study. The experimental data followed a normal distribution and were expressed as the mean ± standard deviation. Univariate analysis of variance was used for univariate design and comparisons of measurement data among multiple groups. The LSD method was used for multiple comparisons. *P* < .05 was considered statistically significant. Results were graphically presented using GraphPad prism 8.0 software.

## 3. Results

### 3.1. SAN treatment NPC target screening and protein interaction network

**Figure 1.**
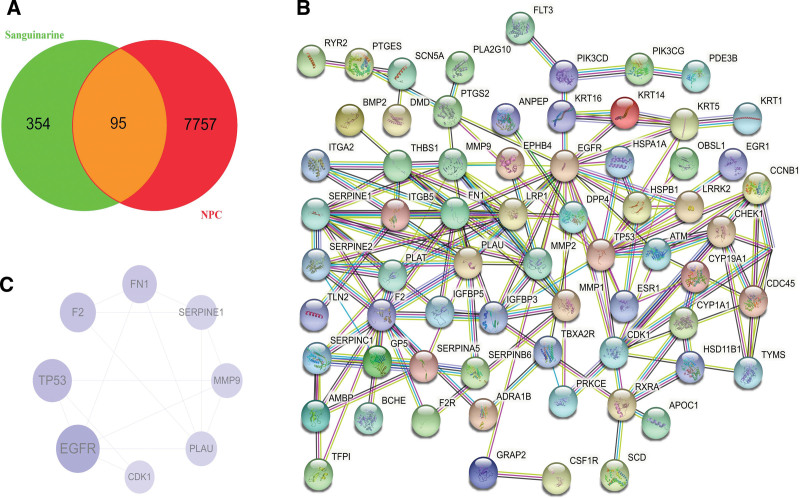
Venn diagram of active component targets of Chelidonium majus and nasopharyngeal carcinoma (NPC) disease targets (A). Common target interaction network (B). Core targets of SAN anti-NPC (C).

A total of 7852 disease genes were extracted from the GEO database. Drug targets were searched in the SwissTargetPrediction database. A total of 449 SAN targets were obtained by removing duplicates and corrected using the Uniprot database. As a result, 95 intersection targets were identified (Fig. 1A). At the same time, function-related PPI data for these 95 targets were collected via the STRING database to construct a SAN treatment NPC target and its function-related protein interaction network, with a total of 102 nodes and 175 edges (Fig. 1B).

### 3.2. PPI network topology parameter analysis and core target screening

The mapped intersection protein was introduced into the Cytoscape 3.7.1, and the topological parameters of SAN anti-NPC target and function-related protein interaction network were calculated. The median degree of freedom for the target was 4.588, and the maximum degree of freedom was 19. Therefore, the range of core target screening conditions was set to 10–19, and 8 core targets were obtained, including EGFR, TP53, F2, FN1, PLAU, MMP9, SERPINE1, and CDK1 (Fig. 1C).

### 3.3. GO and KEGG pathway enrichment analyses of core targets

**Figure 2.**
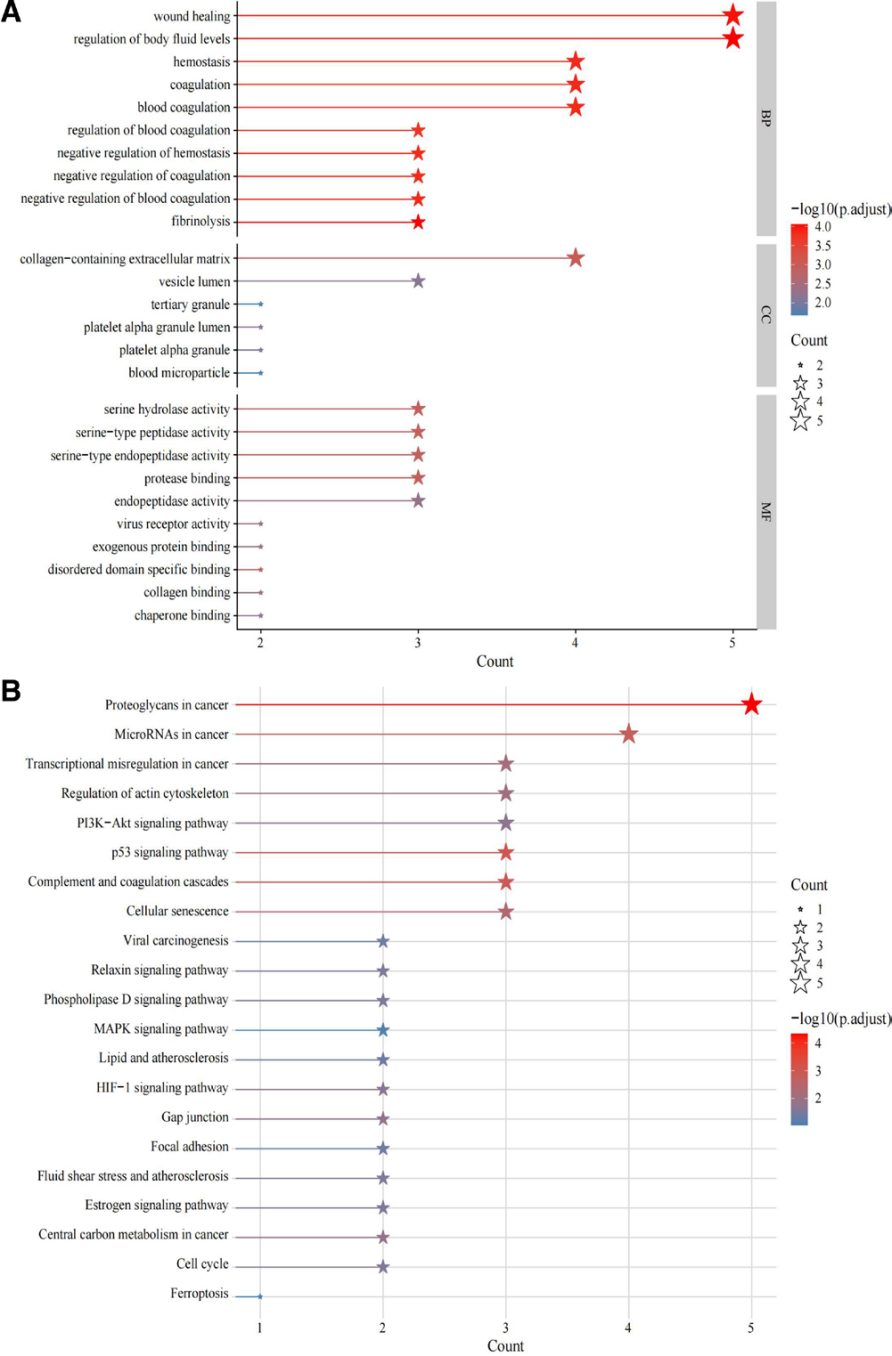
GO function (A) and KEGG enrichment (B) analyses.

A total of 530 GO items were obtained via GO biological process analysis of core targets. GO classification and enrichment analysis showed that cell cycle checkpoint signaling, negative regulation of cell cycle phase transition and cell cycle process in BP analysis, mitochondrial matrix and cyclin-dependent protein kinase holoenzyme complex in CC analysis, histone kinase activity, and cyclin-dependent protein serine/threonine kinase regulator activity in MF analysis had a higher enrichment proportion and lower *P* value (Fig. 2A). KEGG pathway enrichment analysis resulted in 42 KEGG items. SAN anti-NPC target genes were mainly involved in the PI3K/AKT signaling, MAPK signaling, and p53 signaling pathways (Fig. 2B).

### 3.4. Molecular docking

**Figure 3.**
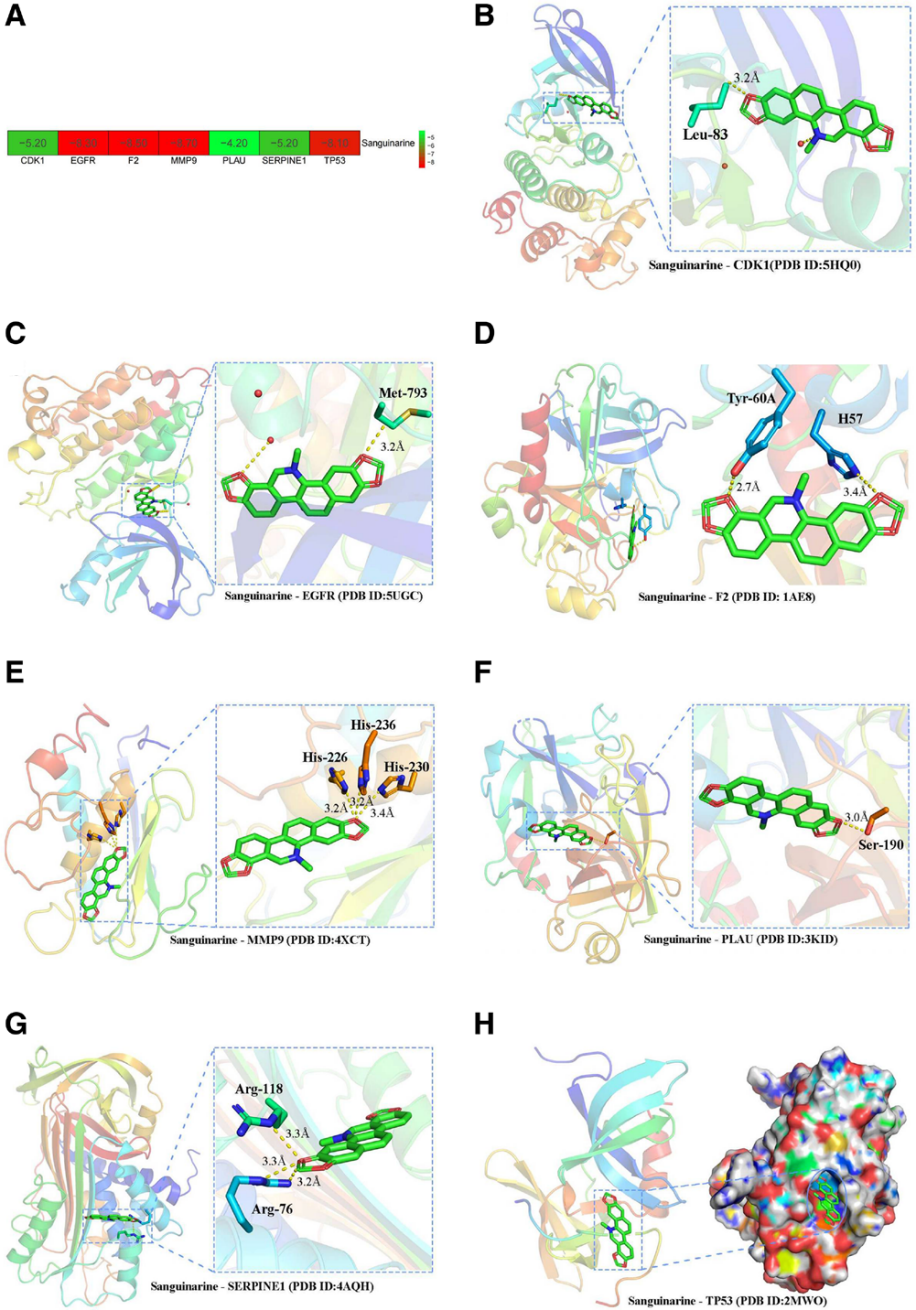
Molecular docking analysis of protein binding ability for SAN. Protein binding energy for SAN (A); binding sites between SAN and core targets (B: CDK1; C: EGFR; D: F2, E: MMP9; F: PLAU; G: SERPINE1; and H: TP53).

Search for proteins related to 8 core targets through the PDB database established a suitable active cavity box model and then docked with compound SAN. Figure 3 shows that the binding energies of EGFR (PDB ID:5UGC), TP53 (PDB ID:2MWO), F2 (PDB ID: 1AE8), PLAU (PDB ID:3KID), MMP9 (PDB ID:4XCT), SERPINE1 (PDB ID:4AQH), and CDK1 (PDB ID:5HQ0) to SAN were all <0 kcal/mol, indicating that these core targets had good affinity and good binding to SAN. FN1 did not result in a suitable protein for molecular docking.

### 3.5. Experimental results

#### 3.5.1. SAN inhibits proliferation of NPC cells.

**Figure 4.**
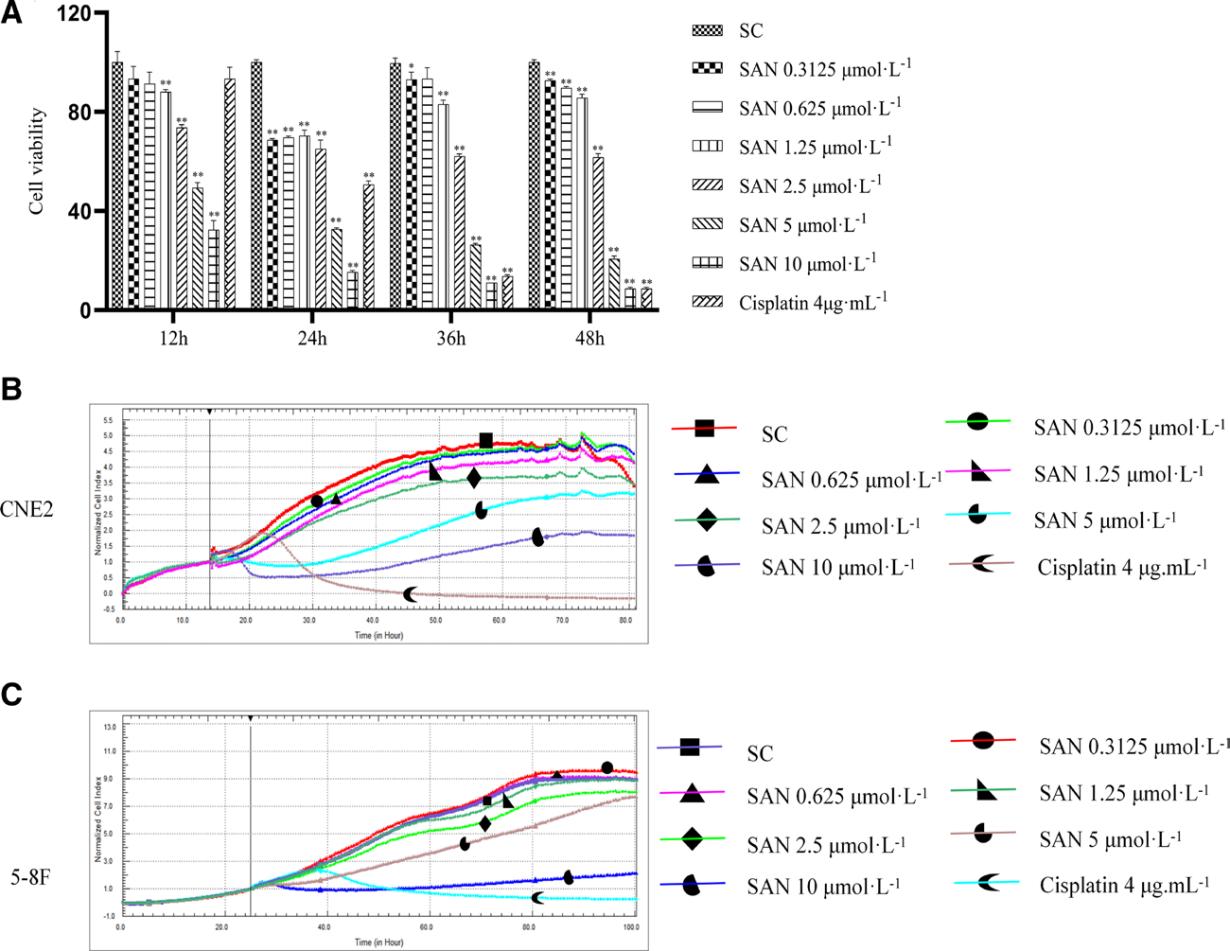
MTT assay results for the effects of different SAN concentrations (0.3125, 0.625, 1.25, 2.5, 5, and 10 μmol·L^−1^) on proliferation activity of CNE2 cells (compared to solvent group, *P < .05, **P < .01) (A); RTCA of the effects of different SAN concentrations on proliferation activity of CNE2 (B) and 5-8F (B) cells.

**Figure 5.**
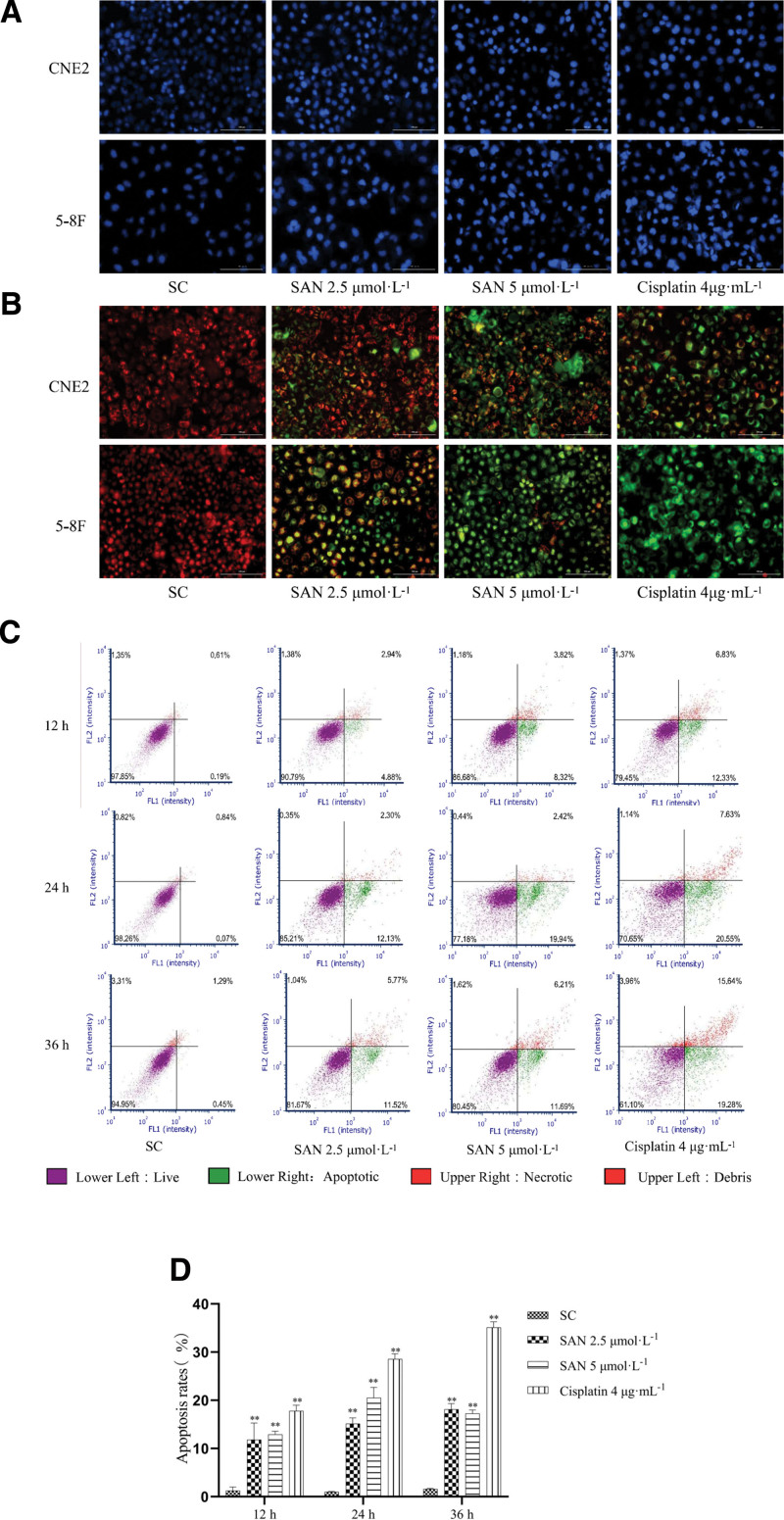
Apoptosis-inducing effect of SAN on NPC cells determined using Hoechst 33342 staining showing cellular nuclei (blue) (A), cellular membrane potential (B) detected using JC-1, and apoptosis rate via annexin V-FITC/PI double fluorescence staining (C, D) (bar = 100 μm, 200×; compared to solvent control group,**P < .01).

After treatment with different concentrations (0.3125, 0.625, 1.25, 2.5, 5, and 10 μmol·L^−1^) of SAN and Cis (4 μg·mL^−1^) for 12, 24, 36, and 48 hours, the proliferation of CNE2 cells was significantly inhibited (*P* < .05 and *P* < .01, respectively; Fig. 4A). RTCA results also showed that SAN and Cis significantly inhibited the proliferation of CNE2 (Fig. 4B) and 5-8F cells (Fig. 4C).

#### 3.5.2. NPC cell apoptosis induced by SAN.

After 36 hours of SAN treatment, Hoechst 33342 staining indicated that nuclei in the control group appeared to be of uniform light blue color, whereas nuclei in the drug group appeared to be bright blue, with nuclear pyknosis and transformation (Fig. 5A). Similarly, JC-1 staining indicated that the cell membrane in the control group was red, while the green fluorescence level in the drug group gradually increased, indicating that SAN reduced the membrane potential of CNE2 and 5-8F cells (Fig. 5B). Annexin V-FITC/PI double fluorescence staining results showed that SAN increased the apoptosis rate of NPC cells after 12, 24, and 36 hours of treatment (Fig. 5C and D).

#### 3.5.3. Effect of SAN on apoptosis-related proteins in NPC cells.

**Figure 6.**
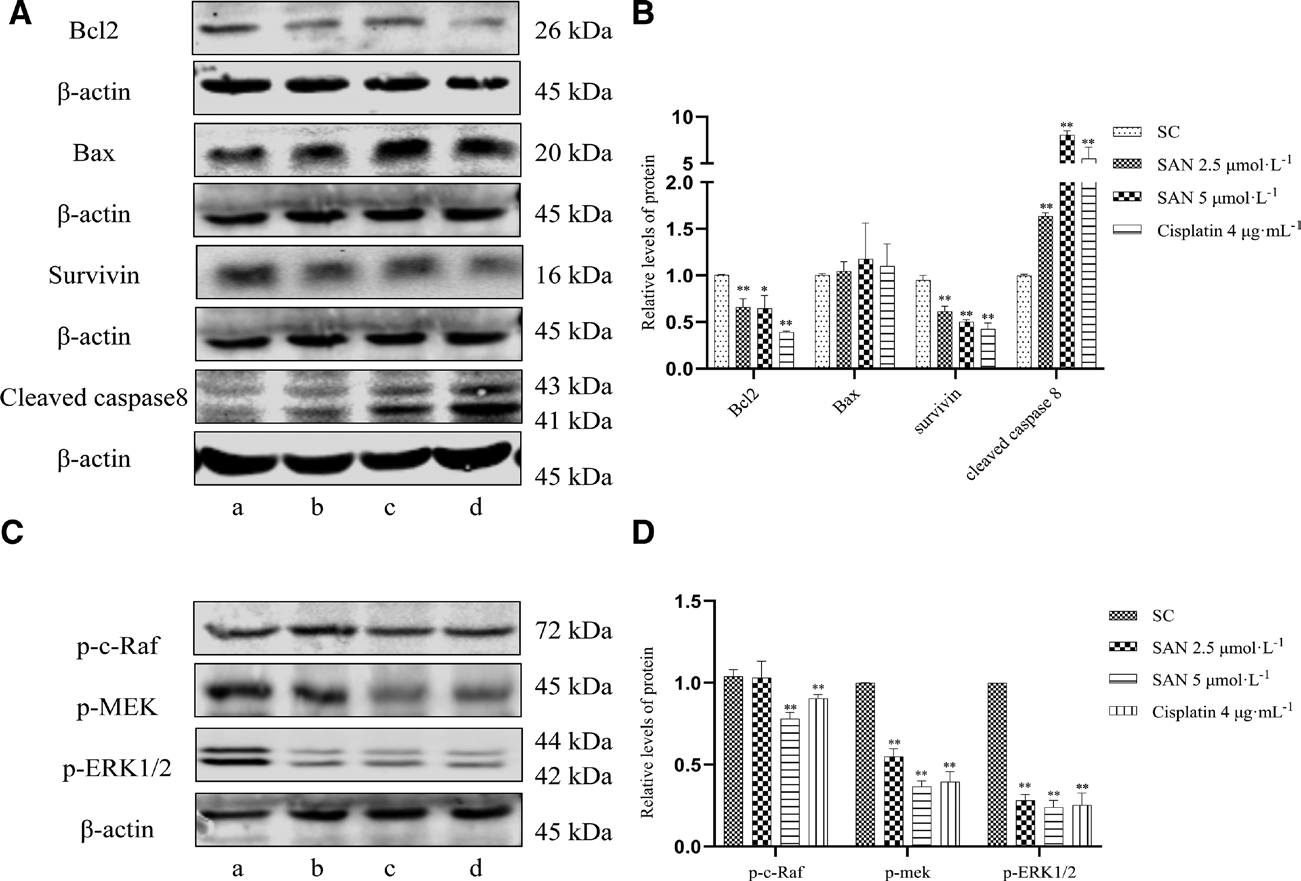
Western blotting was used to determine the effects of SAN on apoptosis-related and key proteins in MAPK/ERK1/2 signaling pathway in NPC cells. SAN effects on expression levels of Bcl2, survivin, Bax and cleaved caspase-8 (A, B). Effect of SAN on levels of p-c-Raf, p-MEK, and p-ERK1/2 (C, D; compared to solvent control group,*P < .05, **P < .01). a:SC;b:SAN 2.5 μmol·L^-1^;c:SAN 5 μmol·L^-1^;d:Cisplatin 4 μg·mL^-1^

After 36 hours of drug treatment, the expression levels of antiapoptotic proteins Bcl2 and survivin in the SAN group decreased, while the expression levels of proapoptotic protein cleaved caspase-8 increased compared to the levels in the control group. Furthermore, the expression level of Bax in the drug group increased, although this result was not statistically significant compared to the control group (Fig. 6A and B). These results suggest that the SAN-induced apoptosis of NPC cells may occur via regulation of Bcl2, survivin, Bax, and cleaved caspase-8.

#### 3.5.4. Effect of SAN on MAPK/ERK1/2 signaling pathway in NPC cells.

Compared to the control group, SAN decreased the expression levels of p-c-Raf, p-mek, and p-ERK1/2, which are key proteins in the MAPK/ERK1/2 signaling pathway (Fig. 6C and D). These results suggest that SAN can reduce the activity of the MAPK/ERK1/2 signaling pathway in NPC cells.

## 4. Discussion

NPC is an epithelial malignant tumor, and its incidence is increasing every year. It has the characteristics of regional and ethnic distribution, especially in South China, Southeast Asia, and other locations.^[25,26]^ At present, radiotherapy and chemotherapy are the main treatments for NPC. Although traditional chemical drugs are effective, they are toxic, have side effects, and are likely to result in drug resistance development. Therefore, it is of great significance to research and develop new drugs for the treatment of NPC. SAN is a phenanthrene alkaloid. Compared to synthetic drugs, SAN has the characteristics of low toxicity, has fewer adverse reactions, and demonstrates high bioavailability and reliable efficacy. SAN has been shown to have significant antitumor activity,^[12,15,27,28]^ which is related to cell cycle inhibition and cell apoptosis induction and affects the signal transduction pathway and inhibits angiogenesis.^[29]^ Therefore, the present study used network pharmacology to explore the target of SAN against NPC in order to reveal its mechanism and provide a scientific basis for its further clinical application.

In this study, 95 SAN targets against NPC were obtained based on the reverse targeting strategy. Eight core targets were identified via topological analysis, including EGFR, TP53, F2, FN1, PLAU, MMP9, SERPINE1, and CDK1. Recent studies have shown that EGFR plays an important role in promoting cell proliferation, cell protection, and apoptosis-mediated differentiation, and that EGFR expression is closely related to NPC prognosis.^[30]^ The TP53 gene in NPC is concerning, as the TP53 mutation accounts for only 7% of primary tumors, but the frequency of recurrent NPC is twice as high as that of primary NPC, which has a protective effect on NPC cells.^[31]^ The expression of fibronectin1 (FN1), MMP9, and SERPINE1 was positively correlated with NPC metastasis and promoted the proliferation and invasion of NPC cells.^[32–34]^ CDK1 plays an important role in the development of NPC.^[35]^ The results of molecular docking showed that there is good binding between SAN and the core targets, suggesting that the anti-NPC effect of SAN is related to the regulation of these core targets. The present study experiments showed that SAN can inhibit the proliferation and induce apoptosis of NPC cells. Bax and Bcl2 are the key factors determining apoptosis in tumor cells.^[36]^ The present results showed that SAN can reduce the expression of antiapoptotic protein Bcl2 and increase the expression level of proapoptotic protein Bax. Caspase-8 mainly mediates the mitochondrial apoptosis pathway. Prabhu et al and others have shown that SAN can promote the expression of cleaved caspase-8 and stimulate mitochondrial apoptosis in non-small cell lung cancer.^[12]^ Similarly, the present study experiments showed that SAN can increase the expression of cleaved caspase-8 in NPC cells, suggesting that SAN-induced apoptosis in NPC cells may be related to the mitochondrial pathway.

GO enrichment analysis resulted in 530 items, and KEGG pathway enrichment analysis showed 42 items, which were mainly related to the PI3K/AKT, MAPK, and p53 signaling pathways. The MAPK signaling pathway is involved in the occurrence and development of NPC. The effect of SAN on the MAPK/ERK signaling pathway was verified and revealed that SAN can inhibit the expression level of the key proteins in the signaling pathway, including p-c-Raf, p-MEK, and ERK1/2, which was consistent with previous studies,^[37]^ indicating that SAN can inhibit proliferation and induce apoptosis of NPC cells via the MAPK/ERK signaling pathway.

The above analysis and argument indicate that sanguine is suitable for the preparation of anti-nasopharyngeal carcinoma drugs. Although the inhibitory effects of sanguine on nasopharyngeal carcinoma proliferation and induction of apoptosis have been clarified, the process of inducing apoptosis is still unclear. And this study has only completed network pharmacology and cell experiments, therefore, in further research, it is necessary to improve in vivo experiments and the entire regulatory network.

## 5. Conclusion

SAN can inhibit the proliferation and induce the apoptosis of NPC cells via the MAPK/ERK signaling pathway. The core targets of this mechanism action include EGFR, TP53, F2, FN1, PLAU, MMP9, SERPINE1, and CDK1.

## Author contributions

**Data curation:** Wen-Qing Zhang, Ting Lin.

**Formal analysis:** Fang-Liang Zhou.

**Funding acquisition:** Jing-Ying Fan.

**Methodology:** Ying-Chun He, Hong-Jian Shi.

**Project administration:** Ying-Chun He.

**Resources:** Xi-Ran Hu.

**Writing – original draft:** Jing-Ying Fan, Jie Liu.

**Writing – review & editing:** Le Tang, Hong-Jian Shi.
